# Nested PCR Biases in Interpreting Microbial Community Structure in 16S rRNA Gene Sequence Datasets

**DOI:** 10.1371/journal.pone.0132253

**Published:** 2015-07-21

**Authors:** Guoqin Yu, Doug Fadrosh, James J. Goedert, Jacques Ravel, Alisa M. Goldstein

**Affiliations:** 1 Division of Cancer Epidemiology and Genetics, National Cancer Institute, National Institutes of Health, Bethesda, Maryland, United States of America; 2 Institute of Genomic Sciences, University of Maryland School of Medicine, Baltimore, Maryland, United States of America; University of Naples Federico II, ITALY

## Abstract

**Background:**

Sequencing of the PCR-amplified 16S rRNA gene has become a common approach to microbial community investigations in the fields of human health and environmental sciences. This approach, however, is difficult when the amount of DNA is too low to be amplified by standard PCR. Nested PCR can be employed as it can amplify samples with DNA concentration several-fold lower than standard PCR. However, potential biases with nested PCRs that could affect measurement of community structure have received little attention.

**Results:**

In this study, we used 17 DNAs extracted from vaginal swabs and 12 DNAs extracted from stool samples to study the influence of nested PCR amplification of the 16S rRNA gene on the estimation of microbial community structure using Illumina MiSeq sequencing. Nested and standard PCR methods were compared on alpha- and beta-diversity metrics and relative abundances of bacterial genera. The effects of number of cycles in the first round of PCR (10 vs. 20) and microbial diversity (relatively low in vagina vs. high in stool) were also investigated. Vaginal swab samples showed no significant difference in alpha diversity or community structure between nested PCR and standard PCR (one round of 40 cycles). Stool samples showed significant differences in alpha diversity (except Shannon’s index) and relative abundance of 13 genera between nested PCR with 20 cycles in the first round and standard PCR (P<0.01), but not between nested PCR with 10 cycles in the first round and standard PCR. Operational taxonomic units (OTUs) that had low relative abundance (sum of relative abundance <0.167) accounted for most of the distortion (>27% of total OTUs in stool).

**Conclusions:**

Nested PCR introduced bias in estimated diversity and community structure. The bias was more significant for communities with relatively higher diversity and when more cycles were applied in the first round of PCR. We conclude that nested PCR could be used when standard PCR does not work. However, rare taxa detected by nested PCR should be validated by other technologies.

## Background

Polymerase chain reaction (PCR) amplification of 16S rRNA genes followed by next generation sequencing to characterize microbial communities is now the norm in the fields of human health and environmental sciences. The research on human microbial communities has focused on material such as feces, oral and vaginal swabs that have relatively large bacterial populations and few human cells [1,2]. Bacterial communities from body sites such as lung, esophagus and stomach are more difficult to assess by standard PCR due to sparser bacterial populations and relatively high abundance of human DNA (e. g. from human tissue biopsies). Nested PCR is thus necessary for studies on certain human tissue microbiota since it can amplify the target DNA with concentrations several-fold lower than standard PCR [3–7]. Nested PCR involves two rounds of PCR reactions with the first round targeting a wide DNA region and the second round targeting a narrower sub-region of the products of the first round which is used as a template. Barcoded primers have a low efficacy for binding and amplification when targeted sequences are sparse. In nested PCRs, barcoded primers are only used in the second round of PCR when the template is a 100% match and in higher abundance, and amplify the target with greater efficacy. Therefore, nested PCR works for the samples with sparse targets while standard PCR does not. The drawback to the nested PCR is that the bias due to preferential amplification may be greater when two successive PCR reactions are applied [8]. To date, the potential bias of nested PCR combined with next generation sequencing technologies on the interpretation of microbial diversity and structure has not been rigorously examined.

In this study, we evaluated the bias of nested PCR in estimating microbial diversity and community structure by comparing it with standard PCR. We investigated the influence of microbial diversity (relatively low vs. high) and the number of cycles in the first and second round of nested PCR.

## Methods

### Sample collection

Vaginal swabs (17 samples) and fecal samples (12 samples) were collected from adults as previously described [9,10]. The study of vaginal swab samples was approved by the Institutional Review Board at the University of Maryland Baltimore. The study of fecal samples was approved by the National Cancer Institute Special Studies Institutional Review Board. All participants provided written informed consent.

### DNA extraction, PCR amplification and sequencing of 16S rRNA

The DNA from previously published studies was used [9,11]. The designs for nested and standard PCRs are shown in Table 1 [12]. All PCRs had 40 cycles in total. The nested PCRs had either 10 or 20 cycles for the first round of amplifications, with 30 or 20 cycles, respectively, for the second round. These are referred to as Nested_10–30 and Nested_20–20, respectively. For comparison, each nested PCR had a standard PCR with either 10–30 or 20–20 cycles, thereby mimicking the nested PCR cycles but using only primer pair 2. These are referred to as Standard_10–30 and Standard_20–20, respectively. An additional Standard_40 PCR used 40 cycles with primer pair 2.

**Table 1:** The details for nested and standard PCR design and number of successful PCR reactions in vagina and stool samples.

		**No. of successful PCR reactions**	
**PCR name**	**PCR design**	**vagina**	**stool**
**nested PCRs**			
Nested_10–30	10 cycles by primer pair 1, then use the products for 30 cycles by primer pair 2	17	6
Nested_20–20	20 cycles by primer pair 1, then use the products for 20 cycles by primer pair 2	16	12
**standard PCRs**			
Standard_10–30	10 cycles by primer pair 2, then use the products for 30 cycles by primer pair 2	17	11
Standard_20–20	10 cycles by primer pair 2, then use the products for 30 cycles by primer pair 2	17	12
Standard_40	40 cycles by primer pair 2	12	12

Note, Primer pair 1 is 515F and 1492R; primer pair 2 is the tagged 515F and 806R.

For the nested PCRs, primer pair 1 for the first round of amplifications was 515F: GTGCCAGCMGCCGCGGTAA, 1492R: TACCTTGTTACGACTT, and primer pair 2 for the second round was tagged 515F (*AATGATACGGCGACCACCGAGATCTACAC TATGGTAATT* GT GTGCCAGCMGCCGCGGTAA, tagged 806R *CAAGCAGAAGACGGCATACGAGAT XXXXXXXXXXXX AGTCAGTCAG CC* GGACTACHVGGGTWTCTAAT). For standard PCR, only primer pair 2 was used. The italic parts of primer pair 2 were the Illumina adapter, primer pad and linker. All PCRs were performed in a reaction mixture (25 μL) containing 1 μL of dissolved DNA (10 ng), 0.5 μl of each primer (10 μM concentration), 12.5 μl 2x Phusion Mastermix with HF Buffer, 0.75 μl DMSO and water. The amplification program used was as follows: 98°C for 1 min; number of cycles of 98°C for 15 s, 55°C for 15 s, and 72°C for 30 s; and 72°C for 1 min. For nested PCR and the standard PCRs that mimic nested PCRs, the first round of PCR products were cleaned up using ExoSAP-IT according to the manufacturer’s recommendations. The PCR products for sequencing were cleaned up and normalized using the SequalPrep DNA Normalization Kit (Invitrogen) according to the manufacturer’s recommendations and eluted in 20 μl elution buffer.

Sequencing was performed on an Illumina MiSeq platform using the 250 bp paired end protocol. The details were described previously [12]. The raw sequence data were deposit in Short Sequence Archive database at National Center for Biotechnology Information (Accession, SRP058353).

### Analysis of 16S rRNA gene sequence data

We removed low quality reads (reads with average quality less than 20 over 30bp window based on Phred algorithm; paired reads which have at least one with length less than 75% of its original length) and chimera reads (by UCHIME), and clustered the remaining reads into species-level Operational Taxonomy Units (OTUs) at 97% identity by Usearch method in software package Quantitative Insights into Microbial Ecology (QIIME 1.8.0) [13]. Reads without 97% identical reference in Greengenes database (release gg_13_8, http://qiime.org/home_static/dataFiles.html) were excluded. OTUs with only one read or in only one sample were excluded. Of the reads that passed QIIME’s quality filters, 3,054,521 reads from vagina samples (97% of the filtered reads) and 1,363,608 reads from stool samples (91% of the filtered reads) hit a reference sequence with at least 97% identity. In order to exclude the effect of extremely rare OTUs, we also used more strict filtering criteria. The OTUs with less than 20 reads or in less than 10% of samples were excluded. Of the reads that passed the strict criteria, 3,046,632 reads from vaginal samples and 1,356,860 reads from stool samples hit a reference sequence with at least 97% identity.

In order to minimize the difference in microbial diversity estimation due to sequencing effort, a random sample of the same sequence depth was drawn 20 times without replacement from each sample. Each participant’s alpha diversity measures were based on the mean values from 20 such random samplings. The minimum sequence depth used for vagina and stool samples were 16,279 and 12,746 reads per sample respectively, and 16,214 and 12,377 per sample respectively based on the strict OTU filtering criteria.

Four measures of alpha diversity including number of OTUs, Chao1, Shannon’s Index, and Phylogenetic distance (PD) whole tree were calculated for both less strict and strict OTU filtering criteria. The total number of unique OTUs does not take relative abundance of OTUs into account. Chao1 is bias-corrected for singleton OTUs [14]. Shannon’s index is defined as (negative) the sum over OTUs of the product of the relative abundance of the OTU times the natural logarithm of the relative abundance [15]. PD_whole tree reflects number and relative abundance of OTUs, and phylogenetic divergence among OTUs within an individual [16]. Wilcoxon signed-rank test for paired samples was applied for the difference of alpha diversity between nested PCRs and their controls [17]. Significance level of 0.05 was applied.

A beta-diversity measure, Weighted UniFrac distance matrix, which measures the pairwise difference in microbial diversity among samples, was calculated in QIIME [18]. To provide visualization of the sample distribution patterns, a principal component analysis (PCA) was applied to transform the UniFrac distance matrices into principal coordinates.

Taxonomy was assigned using RDP classifier 2.2 [19]. The relative abundance of bacterial genera was calculated in QIIME [13]. Bray-Curtis dissimilarity matrix (a measure of dissimilarity among community states) and hierarchical clustering with average linkage hierarchical clustering were used to determine community similarity. Linear discriminant analysis (LDA) effect size (LEfSe) method was used to identify genus-level taxa with statistically significant different relative abundance between paired samples amplified by the two different PCR methods [20].

## Results

### Difference in alpha diversity between nested and standard PCR controls

Sequencing 16S rRNA partial genes was successful with at least 16,279 reads (16,214 based on strict OTU filtering criteria) in 79 of 85 (93%) PCRs for the 17 vagina swab samples and with at least 12,746 reads (12,377 based on strict OTU filtering criteria) in 53 of 60 (83%) PCRs for the 12 stool samples. The number of successful PCR reactions for each PCR design is listed in Table 1.

**Fig 1:**
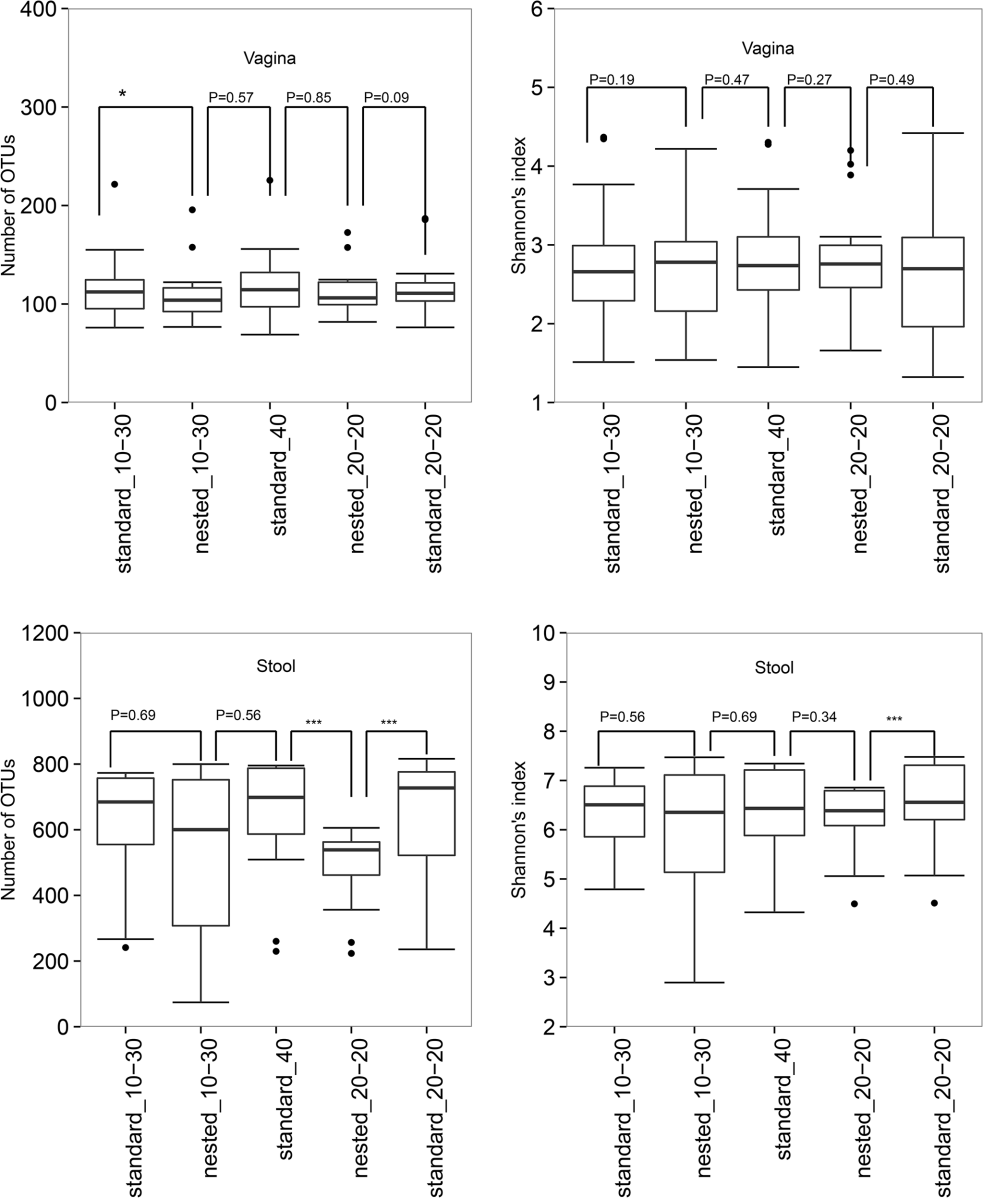
Box plots comparing nested to standard PCRs in number of OTUs and Shannon’s index. Note, *P<0.05, **P<0.01, ***P<0.001, the P values were calculated by the Wilcoxon Matched-Pairs Signed Ranks test.

Compared with their matching standard PCRs, both nested PCRs of vagina samples had on average 3–7% fewer OTUs, 2% lower in Shannon’s index and 2–8% lower in Chao1 and PD_whole tree. The differences by PCR methods were not significant except between Nested_10–30 and Standard_10–30 in number of OTUs (P = 0.02, Fig 1), and Nested_20_20 and Standard_20_20 in PD_whole tree (p = 0.04). Based on our strict OTU filtering criteria, the differences by PCR methods were only statistically significant between Nested_10–30 and Standard_10–30. These two methods differ in Number of OTUs, Chao1 and PD_whole tree (p<0.003), but not in Shannon’s index (S1 Table).

Likewise, both nested PCRs of stool samples had on average 17 to 24% fewer OTUs, 2–8% lower in Shannon’s index and 12–21% lower in Chao1 and PD_whole tree compared with their matching standard PCRs. All differences between Nested_20–20 and standard PCRs were statistically significant (P≤0.01) except for Shannon’s index between Nested_20–20 and Standard_40 (shown in Fig 1 for Number of OTUs and Shannon’s index). We found no statistically significant difference in alpha diversity between Nested_10–30 and the standard PCRs. The results based on strict OTU criteria are the same as the ones based on less strict OTU criteria (not shown).

Unique sets of OTUs were detected only in the nested PCRs or only in the standard PCRs. The discordant OTUs between Nested_10–30 and Standard_40 OTUs are listed in S2 Table. The average counts and sums of relative abundance of these discordant OTUs are shown in Table 2. On average, 34–51 OTUs were only detected in nested PCRs and 43–58 OTUs were only detected in the standard PCRs for vagina samples; 141–187 OTUs were only detected by nested PCR and 223–265 OTUs were only detected by standard PCRs for stool samples. The majority of the discordant OTUs had low or very low abundance (S2 Table). They comprised 30%-47% of the total number of OTUs in vagina and 27–44% in stool samples, but they only contributed 0.2–0.3% of total OTU abundance in the vagina microbiota, and slightly more (0.9–16.7%) in the stool microbiota.

**Table 2:** Summary of number of OTUs which were detected by only either nested PCRs or standard PCR controls when compared with each other for the matched samples.

	****Unique to nested PCRs****			****Unique to standard PCRs****		
****Sample****	****Nested PCR****	****Average number of unique OTUs****	****Sum of relative abundance****	****Standard PCR****	****Average number of unique OTUs****	****Sum of relative abundance****
**Vagina**	10–30	39	0.003	10–30	50	0.004
		51	0.003	40	43	0.003
	20–20	34	0.002	20–20	58	0.003
		48	0.003	40	44	0.003
**Stool**	10–30	141	0.102	10–30	265	0.167
		184	0.058	40	223	0.164
	20–20	160	0.009	20–20	258	0.038
** **		187	0.012	40	225	0.038

### Comparison of beta diversity between nested and standard PCR

**Fig 2:**
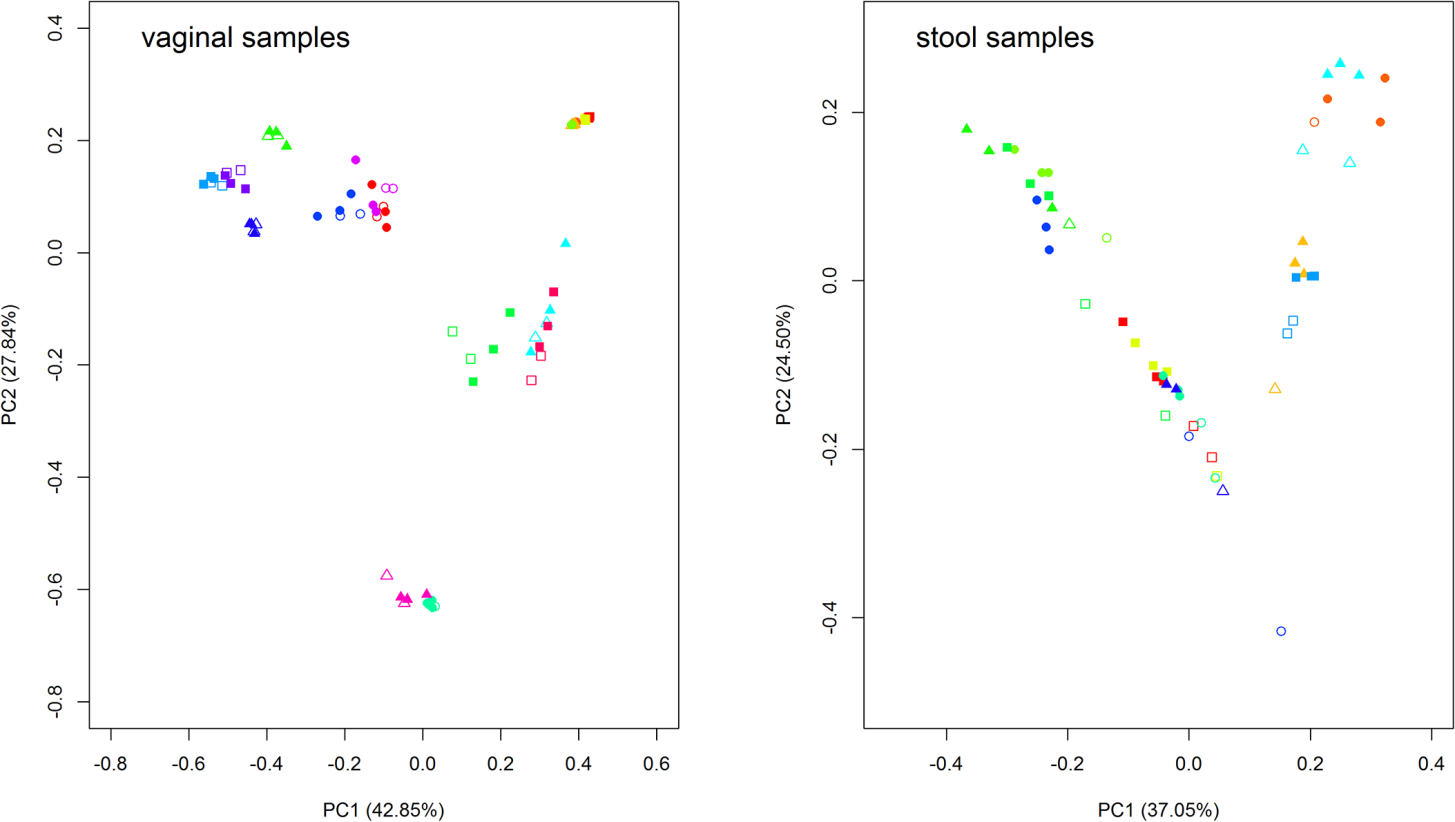
Principal coordinates analysis (PCoA) of weighted UniFrac distance. Proportion of variance explained by each axis is denoted in the corresponding axis labels. Each symbol (designated by the combination of color and shape) represents each subject with the open symbols for the nested PCRs and the closed symbols for the standard PCRs. For example, the blue circles represent subject 1 with open blue ones for two nested PCR results and closed blue ones for three standard PCR results.

**Fig 3:**
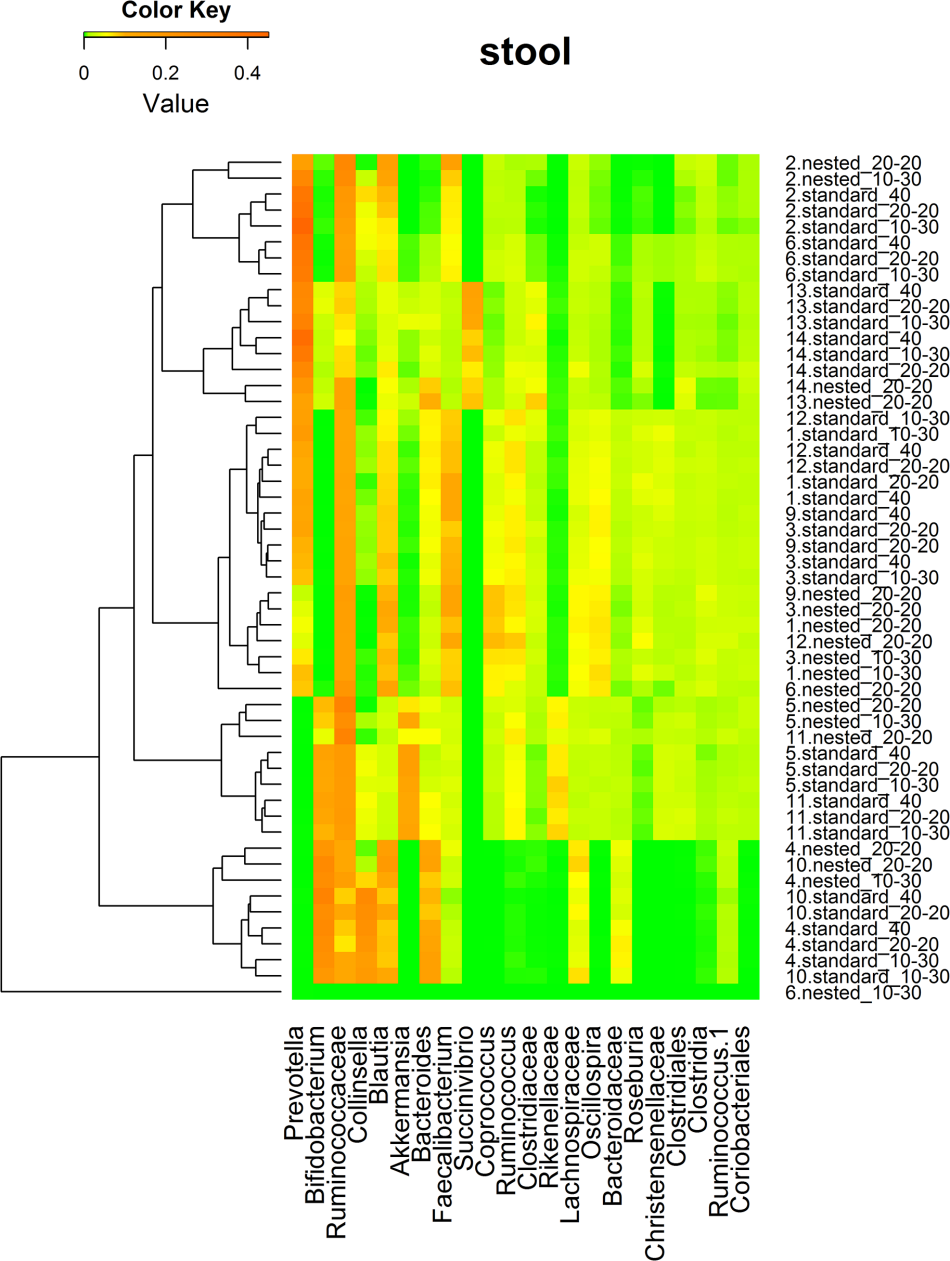
Clusters of stool samples based on bacterial genus relative abundance. Heatmaps were based on the hierarchical clustering solution (Bray-Curtis) distance metric and average clustering method. Row represents different sample ID (The number before the period is the subject ID; The text after the period is the PCR method used.). Columns represent the predominant bacterial genera with mean relative abundance of 0.01 or greater. The colors in the heatmaps represent the relative abundance of each genus, as indicated in the upper left corner of each panel.

Weighted UniFrac-based PCoA plots (Fig 2) revealed a strong pattern of primary clustering of community structure by subject for the vaginal swabs, but not for the stool samples. Samples from the same subject clustered together, and within-subject UniFrac distances were generally smaller than between-subject distances, suggesting the community composition of samples from the same subject were more similar to each other and consisted of bacterial lineages sharing a common evolutionary history. For the stool samples, however, several nested PCRs were outliers, far distant from their standard PCR controls (Fig 2 and Fig 3).

### Relative abundance of taxa at genus level between nested and standard PCRs

**Fig 4:**
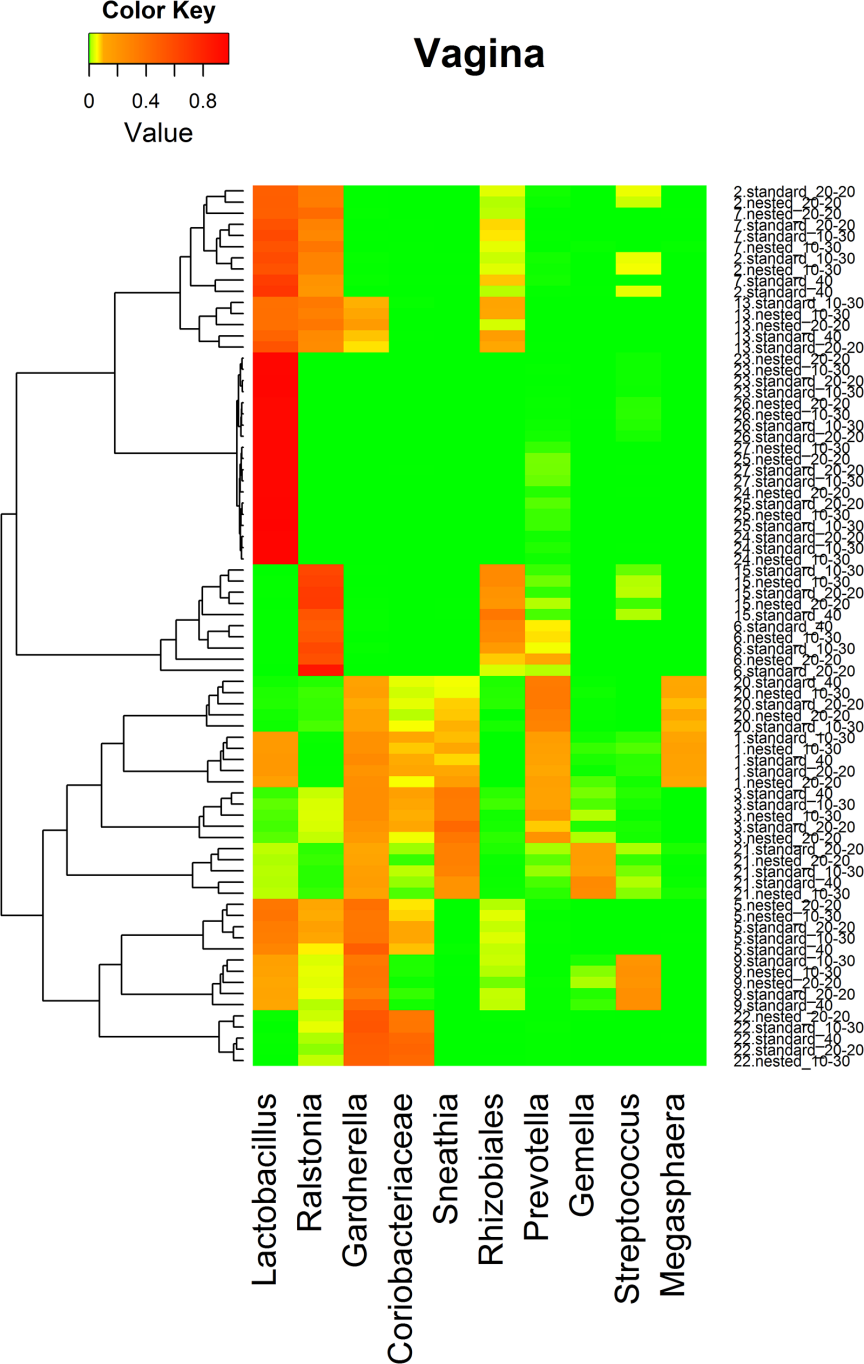
Clusters of vagina samples based on bacterial genus relative abundance. Heatmaps were based on the hierarchical clustering solution (Bray-Curtis) distance metric and average clustering method. Row represents different sample ID (The number before the period is the subject ID; The text after the period is the PCR method used.). Columns represent the predominant bacterial genera with mean relative abundance of 0.01 or greater. The colors in the heatmaps represent the relative abundance of each genus, as indicated in the upper left corner of each panel.

**Fig 5:**
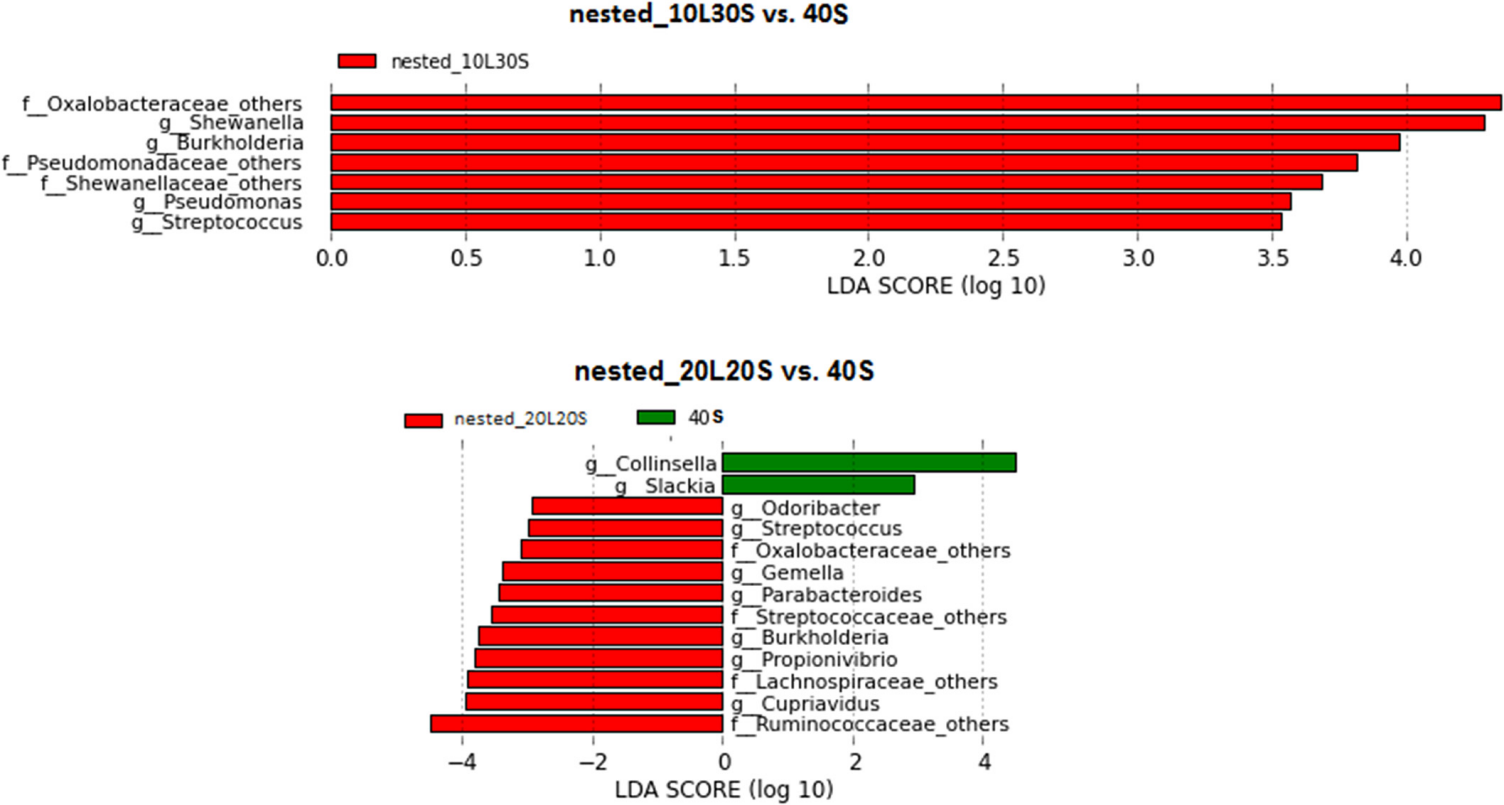
Histogram of the LDA scores computed for microbial genera differentially abundant between nested and standard PCRs in stool samples. The taxa shown in red are the ones with significantly higher abundance by nested PCRs while the taxa shown in green are the ones with significantly higher abundance by standard PCR. The g or f before the taxon name means genus or family.

The heatmap of microbial genus relative abundance (Figs 3 and 4) shows that vaginal samples were clustered by subjects while stool samples were not. Further analysis in LEfSe indicated that no taxon at the genus level was significantly different in relative abundance between nested and standard PCRs for vaginal samples (data not shown). However, compared to Standard_40 in the stool samples, 7 taxa at genus level had higher relative abundance with Nested_10–30 (comprising 1.79% relative abundance). Furthermore, 11 taxa (comprising 7.80% relative abundance) had higher relative abundance and 2 (comprising 1.73% relative abundance) had lower relative abundance with Nested_20–20 than with Standard_40 (Fig 5). Similar distortions (not shown) were also observed between Nested_10–30 and Nested_20–20 compared to their matching standard PCRs.

## Discussion

We evaluated the effect of nested PCR and other factors including cycle number in the first round of nested PCR and community diversity level on measures of microbial community structure. We observed biases by nested PCR in the estimation of microbial diversity and structure both in vaginal and stool samples and the differences were mainly observed between Nested_20–20 and standard PCRs in stool samples.

Our results indicated that nested PCR introduced bias in measurements of community structure, particularly for stool samples. This might be due to the discordant OTUs that were detected only in nested PCR or only in its matching standard PCR. Such discordant OTUs were rare. They contributed 30–47% to the total number of unique OTUs, but were so rare that they contributed only a small proportion (0.002–0.003) of the total abundance of the vaginal microbiota. In the stool samples, which had an average of 631 unique OTUs, discordant OTUs represented 27–44% of the total number of unique OTUs, and they contributed 0.01–0.17 to the total abundance in stool. Alternatively, such unique OTUs might be the true members of the community that could not be consistently amplified due to their rarity or low specificity with the primers used for PCR reactions. Other sensitive and accurate techniques will be required to characterize such rare taxa.

Our results showed significant bias of alpha diversity estimation from standard PCRs by Nested_20–20 but not by Nested _10–30 in stool samples, suggesting that the lower number of cycles in the first round of nested PCR results in less bias. However, since only 6 stool samples for Nested_10–30 were amplified, the low bias in Nested_10–30 might merely reflect limited statistical power. In addition, the total number of cycles might have an effect on the estimation of microbial diversity and structure, which is not estimated in the study.

In conclusion, our results showed that nested PCR will introduce biases in interpreting microbial community diversity and structure with Illumina MiSeq-based 16S rRNA gene profiles. The bias was more significant for high diverse communities or a high number of cycles in the first round of PCR. We suggest that nested PCR should only be applied when standard PCR is unsuccessful, and the number of cycles in the first round of PCR should be as low as possible. Rare taxa detected by nested PCR should be confirmed by other technologies.

## Supporting Information

S1 Table Difference between nested and standard PCRs in vaginal samples. (XLS)

S2 Table List of discordant OTUs between nested_10–30 and standard PCRs; 2a), List of discordant OTUs between nested_10–30 and standard_40 for each vagina samples; 2b), List of discordant OTUs between nested_20–20 and standard_40 for each vagina samples; 2c), List of discordant OTUs between nested_10–30 and standard_40 for each stool samples; 2d), List of discordant OTUs between nested_20–20 and standard_40 for each stool samples (XLS)
